# Obstetric Violence in Spain (Part II): Interventionism and Medicalization during Birth

**DOI:** 10.3390/ijerph18010199

**Published:** 2020-12-29

**Authors:** Desirée Mena-Tudela, Susana Iglesias-Casás, Víctor Manuel González-Chordá, Águeda Cervera-Gasch, Laura Andreu-Pejó, María Jesús Valero-Chilleron

**Affiliations:** 1 Department of Nursing, Faculty of Health Sciences, Universitat Jaume I, 12071 Castellón, Spain; vchorda@uji.es (V.M.G.-C.); cerveraa@uji.es (Á.C.-G.); pejo@uji.es (L.A.-P.); chillero@uji.es (M.J.V.-C.); 2 Department of Obstetrics, Hospital do Salnés, Villgarcía de Aurousa, 36619 Pontevedra, Spain; matronasu@gmail.com

**Keywords:** obstetric violence, Spain, midwife, sexual and reproduction health, medicalization, interventionism

## Abstract

Background: obstetric violence can partially be represented by the high number of interventions and medicalization rates during the birthing process. The objective of the present study was to determine the interventionism and medicalization levels during childbirth in Spain. Methods: a descriptive, retrospective, and cross-sectional study was conducted between January 2018 and June 2019. Results: the intervention percentages were 34.2% for Kristeller maneuver and 39.3% for episiotomy. Differences appeared in public, private, and mixed healthcare settings (*p* < 0.001). The mean satisfaction, with healthcare in the different settings, was estimated at 6.88 points (SD ± 2.146) in public healthcare, 4.76 points (SD ± 3.968) in private healthcare, and 8.03 points (SD ± 1.930) in mixed healthcare (*p* < 0.001). No statistically significant differences were found in Spanish autonomous communities. Conclusions: births in Spain seem to be highly intervened. In this study, a certain equity criterion was found concerning interventionism during childbirth in Spain. Healthcare influenced female intervention, satisfaction, and perception levels for obstetric violence; this evidences that female empowerment plays an important role.

## 1. Introduction

Although no international consensus has been reached regarding a definition of obstetric violence (OV), some Latin American countries have passed laws on this problem [1]. The definition, published in Venezuela in 2007, in the Organic Law on Women’s Right to a Life Free of Violence, defines this concept as “…the appropriation of the body and reproductive processes of women by health personnel, which is expressed as dehumanized treatment, abuse of medication, and converting natural processes into pathological ones, which bring loss of autonomy and the ability to freely decide about their bodies and sexuality, and negatively impact women’s quality of life” [2]. This (and other definitions) refer to abusing medication and interventionism, while giving birth, as OV elements.

A recent literature review classified the unsuitable use of certain procedures and technologies as OV typology [3]. Some of the examples in the review were: iatrogenic procedures, abusive use of oxytocin, being unable to move to bed during childbirth, giving birth in the lithotomy position, routinely performing amniotomy, constant fetal monitoring, women not eating for long periods for no known reasons, unsuitable pain management, not performing skin-to-skin contact, and early umbilical cord clamping [3]. Other classifications exist that indicate excessive or non-consented interventions, as well as medicalization with mistreatment and abuse while giving birth [4].

In 1985, the World Health Organization (WHO)’s Declaration of Strength indicated that all women have the right to suitable prenatal healthcare, and to play a central role in all aspects of this healthcare, including participating in planning, carrying out, and evaluating healthcare [5]. The declaration also indicates the need for competent authorities to prepare specific policies about the use of technology during childbirth, for public and private centers, and to conduct joint surveys to assess healthcare technologies during childbirth [5]. Women formed part of the population to be considered in interviews [5].

Women’s own accounts of childbirth, available in the literature, often describe different obstetric interventions related to OV, such as not being accompanied by anyone, performing unnecessary cesarean sections, routine vaginal palpations, use of oxytocin, or performing the Kristeller maneuver, among other interventions [6,7,8] that are neither recommended nor backed by scientific evidence. Some studies also indicate a lack of respect and a higher level of intervention from the health professional’s point of view [9].

In Venezuela, some constitutive OV actions are considered: making women give birth in the supine position with raised legs; hindering early devotion; denying breastfeeding and skin-to-skin contact; altering the low-risk natural birth process by applying acceleration techniques without obtaining a woman’s voluntary, expressed, and informed consent [2]. In countries such as Brazil, we find that excessive interventions made while giving birth contribute to neonatal/maternal morbidity or mortality [10]. Other studies associate OV perceived by women with the lithotomic position, Kristeller maneuver, while giving birth, denying immediate skin-to-skin contact with their newborn baby [11].

In Croatia, intervention figures represent 54% for the Kristeller maneuver performed during childbirth, 70% for episiotomy, and 78% for using enemas [12]. In Italy, some intervention rates reported during childbirth are striking. Italy presents a rate of 32.54% for cesarean births and 54.24% for episiotomies, plus OV is perceived by 21.2% women [13]. In Spain, very few studies have assessed the interventions and use of technology during childbirth. It is known that the Kristeller maneuver is performed with approximately 25% of women giving birth vaginally [14], even though no scientific evidence exists to support its use. Other interventions, such as no-one accompanying the woman during childbirth, frequent vaginal palpations, using oxytocin, shaving the vulva, or applying episiotomy, are routinely carried out in Spain, and many are not even recorded in women’s medical records [15]. Some reports indicate that cesarean section rates in Spain are high, estimated at roughly 25%, with similar rates for episiotomies and instrumentalized births. Moreover, there is wide variation in Spanish Autonomous Communities (SAC), and between public and private healthcare sectors [16,17].

In Spain, a constitutional right exists that allows free access to the public health system, but the Spanish healthcare management model allows the co-existence of a public healthcare network and privately managed health centers. This means that people who pay for private health insurance can access both public and private healthcare. It is necessary to highlight that a Spanish national survey has reported that 47.3% of those surveyed would choose to pay private insurance, to be attended to during childbirth, or while a family relation does [18].

In 2010, the Spanish Ministry of Health published the Clinical Practice Guidelines for Healthcare during Normal Births, which urged the private health sector to be more transparent regarding birth and maternity healthcare indicators (and not solely reflect cesarean rates) [19]. Yet, since then, very little has been made public about these indicators. For all of these reasons, the objective of the present study was to know the interventionism and medicalization levels during childbirth in Spain, in public, private, and mixed healthcare centers, the distribution by SAC, and how OV is perceived by women.

## 2. Materials and Methods

### 2.1. Design, Population, and Sample

A descriptive, retrospective, and cross-sectional study was conducted from January 2018 to June 2019. The methodology is explained in-depth in the previous publication, Part I [20]. This work analyzed the subsample who gave birth during the study period in Spain. Women who were treated during the 2009 to 2018 period, and who completed the survey, were included in the study. The exclusion criteria included those whose childbirth took place at home or in a hospital outside Spanish territory, and the 80% (or more) of the survey forms that were incomplete. Those surveys completed by women from the Ceuta and Melilla SAC were excluded for not being sufficiently representative, as were those who did not answer the province item. The study was designed in accordance with the principles of the Declaration of Helsinki (charity, no maleficence, autonomy, and justice) and with Spanish Organic Law 03/2018 on Protection Personal Data and Guaranteeing Digital Rights. No personal data, IP address, or email that could compromise the participant’s identity was collected; answering the survey implied giving consent. Participants were informed of these aspects before voluntarily answering the survey.

### 2.2. Data Collection

Data collection took place between February and April 2018 using an ad hoc online survey. Those in charge of handing out surveys to women were healthcare professionals, child rearing associations, breastfeeding support groups, administrators of blogs and the association “El Parto es Nuestro/Birth is Ours” [21]. The link to the survey was forwarded via social networks, such as WhatsApp and Facebook [22,23].

The main study variables were the received healthcare type (public, private, or mixed, understood as women freely choosing between private and public healthcare), and the SAC attended during childbirth. Other variables were: believing they received unnecessary and/or painful interventions (yes, no, do not know); perceiving having suffered OV (yes, no, not know/no answer); feeling satisfied with the received healthcare (visual analogical scale, from 1 not at all satisfied to 10 extremely satisfied); and general view of the received healthcare: (a) empowered and satisfied; (b) insure, vulnerable, guilty, incapable; (c) indifferent; (d) not know/no answer.

Variables related to the interventions that women perceived as being unnecessary during childbirth were added, such as: using cupping glass or forceps, Hamilton maneuver, no one allowed to accompany them, lack of information, shaving vulva, using enemas, not being allowed to eat/drink, limiting movements, amniorrhexis, using oxytocin, constant vaginal palpations, Kristeller maneuver, early umbilical cord clamping, episiotomy, cesarean, manually removing placenta, not allowing skin-to-skin contact, bottle feeding the baby without the mother’s consent, or taking the baby away to perform medical actions. These variables were measured as “yes/no”; more than one variable could be answered.

### 2.3. Statistical Analysis

Data were processed using the Statistical Package for the Social Sciences (SPSS) v. 25, IBM, Armonk, NK, United States of America. A descriptive analysis was done on all of the variables with frequency and percentage. A bivariate analysis with the Chi squared test was carried out using contingency tables to compare the interventions and medicalization of the birth process in public, private, and mixed healthcare centers, and in the national territory, according to the cluster groups that the analysis gave in Part I, where SAC were classified according to how women perceived OV [20]. In this way, the distribution by cluster groups was as follows: group 1 was made up of SAC Madrid, Basque Country, Principality of Asturias and Castilla y León; group 2 with SAC Catalonia, Valencian Community, Aragón and Castilla-La Mancha; group 3 with Andalusia, Balearics, Canaries and Navarre; group 4 with Murcia Region, Galicia, Extremadura and Cantabria; the last group was formed by only one SAC: La Rioja. Women’s satisfaction with the healthcare they received was analyzed by a one-factor ANOVA.

Finally, a binary logistic regression analysis was performed to verify which obstetric interventions were associated independently with the variable “perceived OV”. Statistical significance was set at *p* < 0.05.

## 3. Results

We obtained 17,742 surveys, of which 201 were eliminated (1.13%): 88 (0.49%) for being completed by women who give birth abroad or for not being properly completed; 17 (0.09%) for coming from SAC Ceuta and Melilla; and 96 (0.54%) for not answering the province variable. The final sample comprised 17,541 surveys. Of these, 49.5% (*n* = 8675) of the women negatively answered if they had received unnecessary and/or painful procedures while giving birth, 44.4% (*n* = 7786) reported they had, and 6.2% (*n* = 1080) answered, “don’t know”.

We provide details of the unnecessary and/or painful procedures that women perceived they had undergone (*n* = 8866): 23.6% (*n* = 2094) using cupping glass or forceps; 21.5% (*n* = 1902) Hamilton maneuver; 27.9% (*n* = 2474) no-one accompanied them; 42.1% (*n* = 3735) lack of information; 7.7% (*n* = 687) shaving vulva, 9.1% (*n* = 803) applying enema; 34.3% (*n* = 3043) not being allowed to eat/drink during childbirth; 39.5% (*n* = 3505) restricted movements; 36.3% (*n* = 3216) amniorrhexis; 48.3% (*n* = 4281) using oxytocin; 31.9% (*n* = 2824) constant vaginal palpations; 34.2% (*n* = 3030) Kristeller maneuver; 21.0% (*n* = 1864) early umbilical cord clamping; 39.3% (*n* = 3483) performing episiotomy; 16.9% (*n* = 1502) unnecessary cesarean; 11.2% (*n* = 996) manually removed placenta; 36.9% (*n* = 3274) separating baby for no justified reason; 13.6% (*n* = 1206) bottle feeding the newborn without consent; 32.1% (*n* = 2850) taking the baby away for some test or technique; 10.1% (*n* = 899) other interventions.

### 3.1. Satisfaction and Interventions while Giving Birth and Their Relation to Received Healthcare Type

Of all the women, 65.3% (*n* = 11,450) women were attended to by the public healthcare sector and 24.3% (*n* = 4261) by a mixed public–private healthcare setting. The remaining 10.4% (*n* = 1830) went to a private healthcare center. Women’s satisfaction scored means of: 6.88 points (SD ± 2.146) for public healthcare; 4.76 points (SD ± 3.968) for private healthcare; and 8.03 points (SD ± 1.930) for mixed healthcare. There were statistically significant differences in groups (*p* < 0.001). When examining how they felt about the received healthcare, statistically significant differences were observed for the different healthcare types (*X*^2^ = 1686.89, *df* = 6, *p* < 0.001) (Table 1).

**Figure 1:**
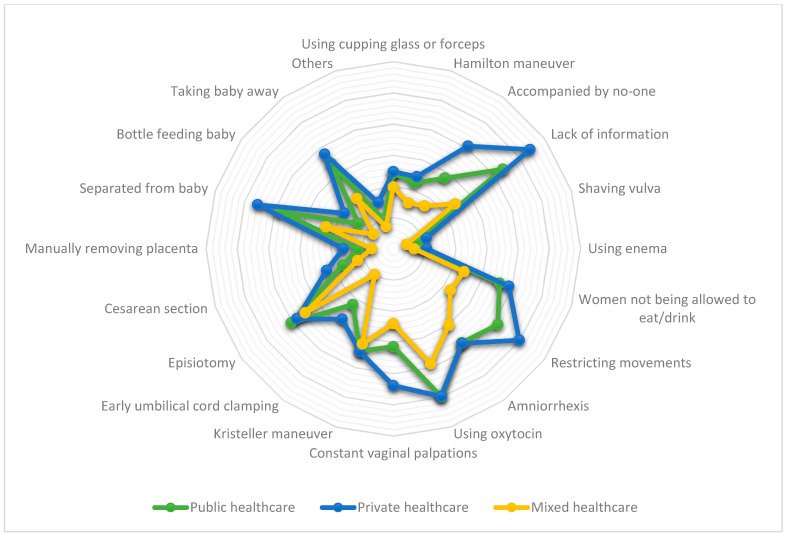
Interventions while giving birth according to received healthcare type.

For healthcare type, 48.6% (*n* = 5561) of the women reported unnecessary and/or painful procedures in public healthcare, 58.4% (*n* = 1069) in private healthcare, and 27.1% (*n* = 7786) in mixed healthcare (*X*^2^ = 850.74, *df* = 4, *p* < 0.001). Of all the women who answered they had, 74.3% (*n* = 5077) also reported perceiving OV (*X*^2^ = 6862.82, *df* = 2, *p* < 0.001). Table 2 shows the analysis of perceiving OV in accordance with having endured unnecessary and/or painful procedures according to healthcare type. Table 3 and Figure 1 depict the descriptive and comparative data of the received interventions and healthcare type.

### 3.2. Interventions While Giving Birth and Their Relation to Cluster Groups

In the cluster groups, the following answered “yes” regarding receiving unnecessary and/painful procedures: 42.8% (*n* = 2930) in cluster 1; 45.3% (*n* = 2318) in cluster 2; 45.0% (*n* = 1281) in cluster 3; 46.5% (*n* = 1223) in cluster 4; 36.2% (*n* = 34) in cluster 5 (*X*^2^ = 23.81, *df* = 2, *p* = 0.002).

**Figure 2:**
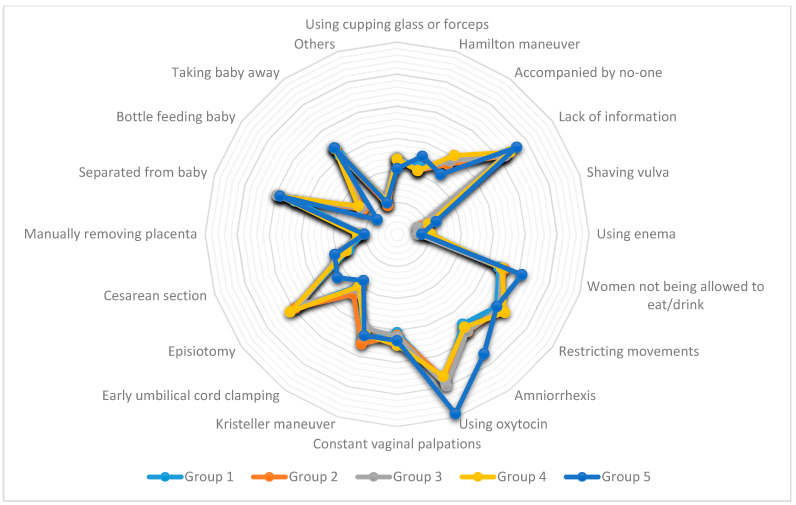
Interventions during childbirth, according to cluster group.

The analysis of interventions per cluster group showed only five interventions (shaving vulva, using enema, Kristeller maneuver, early umbilical cord clamping, and separated from baby) presented statistically significant differences among groups. The descriptive and comparative analysis results, about interventions during childbirth per cluster group, are found in Table 4 and Figure 2.

### 3.3. Obstetric Interventions Related to Women Perceiving OV

The bivariate analysis between interventions and perceiving OV was significant for all of the studied interventions (*p* < 0.001). Finally, when considering the obstetric interventions made during childbirth along with women perceiving OV, a statistically significant logistic regression model was obtained (*n* = 7531, *X*^2^ = 2414.36, *df* = 27, *p* < 0.001). This model explained 39.2% (Nagelkerke R^2^ = 0.392) of variance in perceived OV and correctly classified 78.3% of the cases, with a sensitivity of 54.4% and a specificity of 88.0%. Of all the variables employed as predictors, only the cluster group, using enema, not being allowed to eat/drink, amniorrhexis, and using oxytocin, were not statistically significant (see Table 5).

## 4. Discussion

The present study presents the interventionism and medicalization levels during childbirth in Spain; these levels were assessed in both public and private healthcare sectors. This analysis allowed us to see the interventionism distribution in different SAC by means of previously established cluster groups [20], a certain equity criterion was noted for interventionism and medicalization in the different SAC. Finally, the relation of interventions during childbirth with women’s perceived OV was assessed.

The fact that there is little evidence on interventionism and medicalization levels during pregnancy and childbirth in Spain is worrying. Few reports offer clear information about the rates at which interventions are made during childbirth in Spain. Only a few official reports are available regarding the rates of cesarean sections, perinatal, and/or maternal morbidity or mortality, as valid indicators [24,25], which are practically the only ones found to assess the quality of healthcare received while giving birth in Spain. The present study revealed that the interventionism and medicalization levels of the childbirth process in Spain are high. Techniques that are not recommended by international organizations, such as the WHO, are practiced in Spain. These practices included shaving pubic hair, using enemas, practicing Kristeller maneuver, no-one accompanying women, restricting movements, and lack of information [26]. These techniques are not recommended by the Clinical Practice Guidelines for Healthcare in Spain [15,19], but this seems to make no difference because they continue. Acceptable intervention rates have been set for other techniques to be practiced during childbirth, despite the WHO indicating that setting an acceptable intervention rate is hard with some techniques, such as episiotomy [26]. One technique with a set, suitable intervention rate is the cesarean section. The WHO indicates that its ideal rate must range between 10% and 15% [27]. According to the present study, Spain easily exceeds the recommended rate, and similar data also appear in other reports or studies [15,28].

Certain techniques can have major repercussions on women and a newborn’s health, which is the case with the Kristeller maneuver. This maneuver is neither recommended nor makes the delivery period shorter [19,29]. Its consequences include general bruising, abdominal bruising, fractured ribs, and even uterine tearing [29], which makes the legal repercussions of practicing this maneuver increasingly evident [30]. Notwithstanding, this maneuver is still employed in Spain, and previous reports and studies estimated that it is applied at a rate of around 25% [14,15]. This rate was even higher in the present study, as one third of the women gave a positive response to the question about it. Thus, we reflect that interventions during childbirth can have physical, mental, and emotional repercussions during a woman’s sexual and reproductive life [2,31], and having available, clear evidence for using interventions is essential. Furthermore, this interventionist approach can weaken a woman’s capacity during childbirth [14,32], and have negative effects on her birth experience [33]. It is worth stressing that, while some settings practice a few interventions too late, other women receive too many interventions and too soon [26,34], with possibly fatal consequences for the mother and baby. More studies are required concerning interventions during childbirth, their consequences on the mother’s physical, emotional, and mental health, the possible future conditions for the baby, and the most ideal ways to officially control use of interventions, technologies, and medications while giving birth.

This study determined similar percentages for the interventionism and medicalization rates among SAC in all of the analyzed cluster groups; little variability appeared in birth-related clinical practices. A certain equity criterion was established for interventionism and medicalization during childbirth in Spain. These results differ from previous reports, which indicated that variability in several obstetric interventions made among SAC was present [16,17]. One possible reason for this difference might lie in sources of information, because former reports have taken official medical records and publications as sources to acquire data, while the present study interviewed women, and despite the bias of selection in this study, the findings remain very important. It is true that women’s self-reports can be considered a limitation. Nevertheless, many maneuvers are not recorded in women’s medical records [15]. Given this situation among clusters, female perceptions seem to play a very relevant role in believing they suffered OV or received unnecessary and/or painful interventions. Thus, it is necessary to reflect on the concept that the WHO proposes as a positive delivery experience, which suggests that women wish to physiological labor and birth, and control through involvement in decision making, as well as personal achievements by participating in decision making, even when necessary and desired medical interventions are required [26]. The intention is for any intervention made while giving birth to form part of a security pairing—respect, and good maternal experience—which should be undividable. From this perspective, we ought to bear in mind two important aspects that can promote future works: (a) no available standard or agreement about the OV concept; (b) how the literacy level affects women’s health.

On the OV concept, we found that excessive interventionism and medicalization in physiological processes belong to part of some of their definitions and, hence, this part does not represent a whole. Thus, it seems plausible that, although excessive interventions are representative of the OV concept [35], as our multivariate model demonstrates, they do not represent the whole OV concept. This is why we found some results, such as distributing interventions into cluster groups, which are based on how women perceive OV.

It is possible that some women were unable to identify OV [36], and even take certain obsolete or harmful practices during childbirth as standard practice [10,36,37], which also came across in other areas [38]. Or, perhaps this problem can be more extended than studies actually reveal, and we are currently able to identify only a small part of the problem by taking an iceberg model as a reference, similarly to what other research works have found [39]. This vision invites us to reflect on the literacy concept for health. This concept is defined as the cognitive and social skills that determine motivation and the capacity to access, understand, and employ data that promote and maintain health [40]. Some authors suggest that such literacy includes the social, political and environmental factors that influence health [40]. The differences in the present work among interventions in public/private healthcare, cluster groups, and women perceived having received OV can only be understood from this perspective. As the percentage of OV in Spain is high [20], this perspective can also explain findings, such as this percentage considerably increasing, while bearing in mind the opinions of those women who indicate having suffered unnecessary and/or painful interventions while giving birth, which means that women’s empowerment can play a very important role [41]. Nonetheless, all of these literacy assumptions for health, empowerment, and OV should be confirmed by future studies.

Finally, we ought to focus on the obtained results when comparing healthcare type and obstetric interventions. Apparently, in Spain, a large portion of the population pays for private insurance, to receive the best attention during pregnancy and when giving birth [18]. In international terms, it is worth considering that the private sector attends to a substantial number of women for family planning reasons, such as pregnancies, births, and postpartum periods [42,43]. Thus, as in Spain, in order to improve materno-infant health and well-being, it is important to bear in mind better data collection and minimally controllable public indicators of the materno-infant health services rendered in this sector [19,43]. Furthermore, this study indicates higher interventionism levels in the private sector than in the public one and, in turn, perceived OV is also higher in the private sector [20]. Thus, we should reflect on the technical and human quality of such healthcare in the private health sector, which falls in line with what other authors have reported [44]. This consideration is reinforced by the results obtained for the mixed healthcare type included in the present research work. This mixed type reported a lower interventionism level, more satisfaction, and less perceived OV. It would seem that women’s empowerment plays a fundamental role, as it confers female autonomy to resort to resources and organizations, and to overcome structural or social restrictions [41]. Future studies should assess the use of health services and their type with female empowerment.

This study seems to have correctly assess the interventionism and medicalization phenomenon while giving birth in Spain, by comparing the different, available health sectors in this country (private, public, or mixed). However, this work is not without its limitations, which must be taken into account when interpreting its results. Firstly, we must contemplate that non-probabilistic sampling was carried out, which can affect the sample’s representativeness. A certain selection bias could have come into play as the survey was handed out by groups that might be more sensitive about the studied theme. Some variables were not included, such as age, socioeconomic, and cultural variables, number of children, or date of birth to perform a descriptive sociodemographic analysis in order to make comparisons with other populations. This retrospective study is based on women’s perceived OV, which may lead to memory or information biases. Finally, we stress that the Spanish healthcare model represents a single management model internationally, which means that some of its results cannot be extrapolated to other healthcare systems. Despite all of these limitations, we consider that the findings presented are relevant, offering a global vision of obstetric violence in Spain as a relevant problem that must be addressed by those responsible for the health system.

## 5. Conclusions

Despite its limitations, this study reports relevant results that have not been previously assessed concerning the interventionism and medicalization levels during childbirth in Spain, and their relation to OV. As we found, high levels of intervention can occur during childbirth in Spain, as, among others, maneuvers are applied that can pose a risk for women and babies’ health and lives (such as the Kristeller maneuver). It is very interesting to find that no statistically significant differences appeared among SAC in Spain nationwide, and this interventionism acts as an equity criterion in clinical practice in different SAC.

This interventionism (while giving birth) presents major differences when receiving public, private, or mixed healthcare (understood as that which each woman chooses when being attended to by public or private healthcare). As such, private healthcare has a high interventionism rate, less satisfaction, women feel more insecure and vulnerable, and they perceive more OV. Conversely, mixed healthcare presents lower intervention levels, more satisfaction, and fewer women perceiving OV, which allows us to think that female empowerment plays a very important role. Finally, the logistic regression model shows that most analyzed interventions are representative of OV, without forgetting that interventionism and medicalization during childbirth form only a small part of the OV problem.

## Figures and Tables

**Table 1:** Feelings about the received healthcare depending on healthcare type (*n* = 17,539).

Healthcare Type	Feeling about Received Healthcare Type										
	Empowered and Satisfied		Unsure, Vulnerable, Guilty, Incapable		Do Not Know/No Answer		Indifferent				
	*n*	%	*n*	%	*n*	%	*n*	%	*X* ^2^	*df* ^1^	*p* ^2^
Public	3551	31.0	4728	41.3	839	7.3	2330	20.4	2419.76	6	<0.001
Private	577	31.5	1016	55.5	74	4.0	163	8.9	1089.59	6	<0.001
Mixed	2651	62.2	713	16.7	278	6.5	619	14.5	913.06	6	<0.001
Total	6779	38.7	5467	36.8	1191	6.8	3112	17.7			

^1^ df: Degrees of Freedom; ^2^ Chi square test.

**Table 2:** Descriptive Analysis of perceiving obstetric violence (OV) according to perceiving unnecessary and/or painful procedures and healthcare type (*n* = 15,783).

Unnecessary and/or Painful Procedures	Perceiving OV								
	Public		Private		Mixed				
	Yes % (*n*)	No % (*n*)	Yes % (*n*)	No % (*n*)	Yes % (*n*)	No % (*n*)	*X* ^2^	*df* ^1^	*p* ^2^
Yes	76.1 (3705)	23.9 (1161)	89.8 (906)	10.2 (103)	48.8 (466)	51.2 (489)	461.16	2	<0.001
No	11.2 (538)	88.8 (4266)	5.8 (38)	94.2 (619)	4.3 (121)	95.7 (2670)	114.09	2	<0.001
Do not know	44.1 (220)	55.9 (279)	37.5 (21)	62.5 (35)	24.7 (36)	75.3 (110)	17.95	2	<0.001
Total	43.9 (4463)	56.1 (5706)	56.0 (965)	44.0 (757)	16.0 (623)	84.0 (3269)			

^1^ df: Degrees of Freedom; ^2^ Chi square test.

**Table 4:** Descriptive data of the interventions received during childbirth and the assigned cluster group.

Interventions		Cluster Group												
		1		2		3		4		5				
		*n*	%	*n*	%	*n*	%	*n*	%	*n*	%	*X* ^2^	*df* ^1^	*p* ^2^
Using cupping glass or forceps	Yes	786	23.4	622	23.8	355	23.9	323	23.4	8	20.5	0.46	4	0.977
	No	2569	76.6	1987	73.2	1128	76.1	1057	76.6	31	79.5			
Hamilton maneuver	Yes	749	22.3	544	20.9	311	21.0	288	20.9	10	25.6	2.96	4	0.564
	No	2606	77.7	2065	79.1	1172	79.0	1092	79.1	29	74.4			
Accompanied by no-one	Yes	922	27.5	704	27.0	420	28.3	419	30.4	9	23.1	6.12	4	0.190
	No	2433	72.5	1905	73.0	1063	71.7	961	69.6	30	76.9			
Lack of information	Yes	1414	42.1	1084	41.5	618	41.7	601	43.6	18	46.2	1.89	4	0.756
	No	1941	57.9	1525	58.5	865	58.3	779	56.4	21	53.8			
Shaving vulva	Yes	227	6.8	212	8.1	100	6.7	143	10.4	5	12.8	21.74	4	<0.001
	No	3128	93.2	2394	91.9	13.83	93.3	1237	89.6	34	84.7			
Using enema	Yes	332	9.9	229	8.8	93	6.3	146	10.6	3	7.7	21.06	4	<0.001
	No	3023	90.1	2380	91.2	1390	93.7	1234	89.4	36	92.3			
Women not being allowed to eat/drink	Yes	1122	33.4	920	35.3	509	34.3	476	34.5	16	41.0	2.97	4	0.563
	No	2233	66.6	1689	64.7	974	65.7	904	65.5	23	59.0			
Restricting movements	Yes	1290	38.5	1038	39.8	588	39.6	574	41.6	15	38.5	4.19	4	0.380
	No	2065	61.4	1571	60.2	895	60.4	806	58.4	24	61.5			
Amniorrhexis	Yes	1166	34.8	982	37.6	555	37.4	495	35.9	18	46.2	8.05	4	0.090
	No	2189	65.2	1627	62.4	928	62.6	885	64.1	21	53.8			
Using oxytocin	Yes	1636	48.8	1240	47.5	738	49.8	644	46.7	23	59.0	5.44	4	0.245
	No	1719	51.2	1369	52.5	745	50.2	736	53.3	16	41.0			
Constant vaginal palpations	Yes	1032	30.8	825	31.6	475	32.0	479	34.7	13	33.3	7.16	4	0.128
	No	2323	69.2	1784	68.4	1008	68.0	901	65.3	26	66.7			
Kristeller maneuver	Yes	1161	34.7	941	36.1	459	31.0	454	32.9	13	33.3	12.68	4	0.015
	No	2192	65.3	1668	63.9	1024	69.0	923	67.1	26	66.7			
Early umbilical cord clamping	Yes	664	19.8	604	23.2	312	21.0	277	20.1	7	17.9	11.51	4	0.025
	No	2691	80.2	2005	76.8	1171	79.0	1103	79.9	32	82.1			
Episiotomy	Yes	1321	39.4	1026	39.3	557	37.6	570	41.3	9	23.1	8.52	4	0.074
	No	2034	60.6	1583	60.7	926	62.4	810	58.7	30	76.9			
Cesarean section	Yes	535	15.9	457	17.5	272	18.3	230	16.7	8	20.5	5.46	4	0.243
	No	2820	84.1	2152	82.5	1211	81.7	1150	83.3	31	79.5			
Manually removing placenta	Yes	354	10.6	292	11.2	175	11.8	171	12.4	4	10.3	3.94	4	0.414
	No	3001	89.4	2318	88.8	1308	88.2	1209	87.6	35	89.7			
Separated from baby	Yes	1128	33.6	1017	39.0	584	39.4	530	38.4	15	38.5	26.63	4	<0.001
	No	2227	66.4	1594	61.0	899	60.6	850	61.6	24	61.5			
Bottle feeding baby	Yes	447	13.3	343	13.1	206	13.9	207	15.0	3	7.7	4.24	4	0.374
	No	2908	86.7	2266	86.9	1277	86.1	1176	85.0	36	92.3			
Taking baby away	Yes	1018	30.3	867	33.2	496	33.4	456	33.0	13	33.3	8.09	4	0.088
	No	2337	69.7	1742	66.8	987	66.6	924	67.0	26	66.7			
Others	Yes	325	9.7	246	9.4	175	11.8	149	10.8	4	10.3	7.34	4	0.119
	No	3030	90.3	2363	90.6	1305	88.2	1231	89.2	35	89.7			

^1^ df: Degrees of Freedom; ^2^ Chi square test.

**Table 5:** Odds ratio and 95% confidence intervals of the multivariate logistic regression model that analyzed the obstetric interventions related to perceiving OV (*n* = 7531).

Factors	Wald	OR ^2^ (95% CI)	*p*
Cluster group	2.796	-	0.593
Received healthcare type ^1^	208.594	-	<0.001
Public	102.884	0.451 (0.387–0.526)	<0.001
Private	193.914	0.173 (0.135–0.221)	<0.001
Unnecessary and/or painful procedures ^1^	219.795	0.244 (0.202–0.294)	<0.001
Cupping glass or forceps ^1^	14.63	0.702 (0.585–0.841)	<0.001
Hamilton maneuver ^1^	20.992	0.656 (0.548–0.786)	<0.001
Accompanied by no-one ^1^	25.713	0.639 (0.537–0.759)	<0.001
Lack of information ^1^	131.523	0.420 (0.362–0.487)	<0.001
Shaving vulva ^1^	3.792	0.728 (0.529–1.002)	0.051
Using enema	0.267	1.072 (0.823–1.396)	0.605
Not being allowed to eat/drink	0.007	1.007 (0.858–1.180)	0.936
Restricting movements ^1^	44.978	0.589 (0.505–0.688)	<0.001
Amniorrhexis	1.669	0.907 (0.782–1.052)	0.196
Using oxytocin	0.154	0.971 (0.840–1.123)	0.695
Constant palpations ^1^	46.231	0.577 (0.492–0.676)	<0.001
Kristeller maneuver ^1^	42.399	0.604 (0.519–0.703)	<0.001
Early umbilical cord clamping ^1^	19.402	0.640 (0.525–0.781)	<0.001
Episiotomy ^1^	16.555	0.723 (0.618–0.845)	<0.001
Cesarean section ^1^	11.642	0.701 (0.572–0.860)	0.001
Manually removing placenta ^1^	5.905	0.740 (0.580–0.943)	0.015
Separated from baby ^1^	10.565	0.768 (0.655–0.901)	0.001
Bottle feeding baby ^1^	32.499	0.494 (0.387–0.629)	<0.001
Taking baby away ^1^	5.007	0.833 (0.710–0.978)	0.025
Others ^1^	14.137	0.659 (0.530–0.819)	<0.001

^1^ Variable differs significantly between Obstetric Violence at *p* < 0.05; ^2^ OR: Odds Ratio.

**Table 3:** Descriptive data of the received interventions while giving birth and healthcare type.

Interventions		Received Healthcare Type								
		Public Healthcare		Private Healthcare		Mixed Healthcare				
		*n*	%	*n*	%	*n*	%	*X* ^2^	*df* ^1^	*p* ^2^
Using cupping glass or forceps	Yes	1541	24.2	282	24.7	271	19.8	13.14	2	0.001
	No	4817	75.8	858	75.3	1097	80.2			
Hamilton maneuver	Yes	1413	22.2	277	24.3	212	15.5	36.52	2	<0.001
	No	4945	77.8	863	75.7	1156	84.5			
Accompanied by no-one	Yes	1778	28.0	464	40.7	232	17.0	174.28	2	<0.001
	No	4580	72.0	676	59.3	1136	83.0			
Lack of information	Yes	2760	43.4	640	56.1	335	24.5	270.69	2	<0.001
	No	3598	56.6	500	43.9	1033	75.5			
Shaving vulva	Yes	499	7.8	126	11.1	62	4.5	37.29	2	<0.001
	No	5859	92.2	1014	88.9	1306	95.5			
Using enema	Yes	589	9.3	122	10.7	92	6.7	13.11	2	0.001
	No	5769	90.7	1018	89.3	1276	93.3			
Women not being allowed to eat/drink	Yes	2275	35.8	443	38.9	325	23.8	84.16	2	<0.001
	No	4083	64.2	697	61.1	1043	76.2			
Restricting movements	Yes	2633	41.4	563	49.9	309	22.6	220.02	2	<0.001
	No	3725	58.6	577	50.6	1059	77.4			
Amniorrhexis	Yes	2374	37.3	428	37.5	414	30.3	25.29	2	<0.001
	No	3984	62.7	712	62.5	954	69.7			
Using oxytocin	Yes	3182	50.0	568	49.8	531	38.8	58.11	2	<0.001
	No	3176	50.0	572	50.2	837	61.2			
Constant vaginal palpations	Yes	1995	31.4	500	43.9	329	24.0	114.75	2	<0.001
	No	4363	68.6	640	56.1	1039	76.0			
Kristeller maneuver	Yes	2195	34.5	394	34.7	439	32.1	3.14	2	0.208
	No	4163	65.5	744	65.3	929	67.9			
Early umbilical cord clamping	Yes	1407	22.1	318	27.9	139	10.2	134.32	2	<0.001
	No	4951	77.9	822	72.1	1229	89.8			
Episiotomy	Yes	2571	40.4	435	38.2	477	34.9	15.33	2	<0.001
	No	3787	59.6	705	61.8	891	65.1			
Cesarean section	Yes	1082	17.0	256	22.5	164	12.0	48.52	2	<0.001
	No	5276	83.0	884	77.5	1204	88.0			
Manually removing placenta	Yes	720	11.3	182	16.0	94	6.9	51.75	2	<0.001
	No	5638	88.7	958	84.0	1274	93.1			
Separated from baby	Yes	2442	38.4	521	45.7	311	22.7	161.99	2	<0.001
	No	3916	61.6	619	54.3	1057	77.3			
Bottle feeding baby	Yes	877	13.8	221	19.4	108	7.9	70.56	2	<0.001
	No	5481	86.2	919	80.6	1260	92.1			
Taking baby away	Yes	2150	33.8	428	37.5	272	19.9	117.66	2	<0.001
	No	4208	66.2	712	62.5	1096	80.1			
Others	Yes	618	9.7	178	15.6	103	7.5	48.96	2	<0.001
	No	5740	90.3	962	84.4	1265	92.5			

^1^ df: Degrees of Freedom; ^2^ Chi squared test.

## Data Availability

The data presented in this study are available on request from the corresponding author.
